# Separate neural representations of depression, anxiety and apathy in Parkinson’s disease

**DOI:** 10.1038/s41598-017-12457-6

**Published:** 2017-09-22

**Authors:** Rotem Dan, Filip Růžička, Ondrej Bezdicek, Evžen Růžička, Jan Roth, Josef Vymazal, Gadi Goelman, Robert Jech

**Affiliations:** 1 Edmond and Lily Safra Center for Brain Sciences (ELSC), The Hebrew University of Jerusalem, Jerusalem, Israel; 2 MRI Lab, The Human Biology Research Center, Department of Medical Biophysics, Hadassah Hebrew University Medical Center, Jerusalem, Israel; 3 Department of Neurology and Center of Clinical Neuroscience, First Faculty of Medicine and General University Hospital, Charles University, Prague, Czech Republic; 4 Department of Radiology, Na Homolce Hospital, Prague, Czech Republic

## Abstract

Depression, anxiety and apathy are distinct neuropsychiatric symptoms that highly overlap in Parkinson’s disease (PD). It remains unknown whether each symptom is uniquely associated with a functional network dysfunction. Here, we examined whether individual differences in each neuropsychiatric symptom predict functional connectivity patterns in PD patients while controlling for all other symptoms and motor function. Resting-state functional connectivity MRI were acquired from 27 PD patients and 29 healthy controls. Widespread reduced functional connectivity was identified in PD patients and explained by either the neuropsychiatric or motor symptoms. Depression in PD predicted increased functional connectivity between the orbitofrontal, hippocampal complex, cingulate, caudate and thalamus. Apathy in PD predicted decreased caudate-thalamus and orbitofrontal-parahippocampal connectivity. Anxiety in PD predicted three distinct types of functional connectivity not described before: (i) increased limbic-orbitofrontal cortex; (ii) decreased limbic-dorsolateral prefrontal cortex and orbitofrontal-dorsolateral prefrontal cortices and (iii) decreased sensorimotor-orbitofrontal cortices. The first two types of functional connectivity suggest less voluntary and more automatic emotion regulation. The last type is argued to be specific to PD and reflect an impaired ability of the orbitofrontal cortex to guide goal-directed motor actions in anxious PD patients.

## Introduction

Neuropsychiatric symptoms constitute an integral part of Parkinson’s disease (PD) and include depression, anxiety and apathy^1^. Neuropsychiatric symptoms of PD have considerable impact on quality of life, progression of disability and are associated with negative health outcomes^2^. Cumulative evidence shows that these symptoms are not merely a reaction to psychological distress due to motor disability, but are more likely a direct consequence of the pathology of the disease^3^. Their incidence in PD exceeds the rates in general population as well as in other chronic and/or neurodegenerative diseases^4,5^ with up to 89% of PD patients having at least one symptom^6^.

In addition to their prevalence, depression, anxiety and apathy are highly comorbid in PD. Depression and anxiety were reported to coexist in up to 26% of all PD patients^7^. Apathy shows considerable comorbidity with depression^8^. This complex clinical picture highlights the importance of examining the neurobiological basis of each neuropsychiatric symptom in PD while assessing the presence of all others. Otherwise, findings may be biased due to hidden contributions from other symptoms. However, this is not common practice with no imaging study to date addressing the separate functional neural underpinnings of all the above neuropsychiatric symptoms in PD. In particular, the neural basis of anxiety in PD remains largely unexplored^9^. Moreover, it is unknown whether individual differences in these symptoms are associated with a functional network dysfunction.

To help fill in these gaps, we aimed to determine whether individual differences in each neuropsychiatric symptom among PD patients predict the strength of intrinsic functional connectivity, i.e. the synchronization of BOLD fluctuations between brain regions in the absence of external stimuli, while accounting for the individual differences in all other neuropsychiatric symptoms and motor function. For these purposes, resting-state functional MRI data were acquired from 27 PD patients with varying degrees of depression, anxiety and apathy and a multiple linear regression model was tested. The whole range of individual differences in neuropsychiatric symptoms was considered, from very mild to moderate-severe, obviating the need to define a cutoff score differentiating normal and pathological states. A matched group of 29 healthy control subjects was recruited to evaluate whole-brain functional connectivity patterns associated with PD.

## Results

### Clinical and neuropsychological evaluation

For PD patients, the Beck Depression Inventory, second edition (BDI-II) and Starkstein apathy scores were significantly correlated (r = 0.418; *P* = 0.029) while state anxiety as measured by the Spielberger State-Trait Anxiety Inventory (STAI-state) and BDI-II scores showed a tendency for correlation (r = 0.334; *P* = 0.088). No significant correlation was found for PD patients between Hoehn and Yahr stage or disease duration and any of the neuropsychiatric scales. PD patients and healthy controls differed significantly in STAI-state (*t* = 2.85; *P* = 0.006) and BDI-II scores (*t* = 2.15; *P* = 0.035) with no significant difference in apathy scores (*t* = 0.3; *P* = 0.764).

### Resting state fMRI

#### Depression in PD and functional connectivity

**Figure 1:**
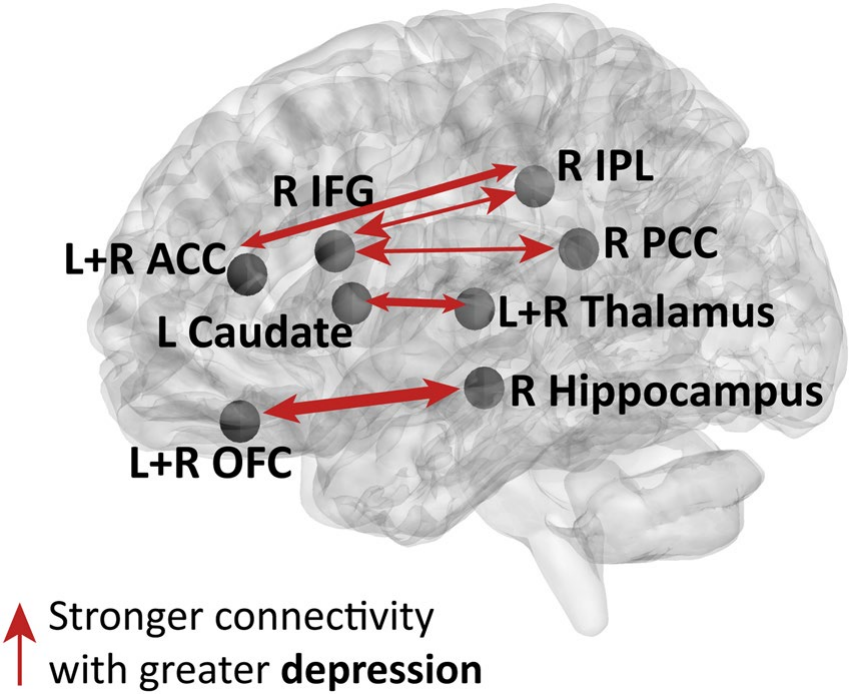
Illustration of functional connections predicted by depression in PD patients. Functional connections predicted by depression while controlling for other neuropsychiatric scales and UPDRS-III are indicated by arrows on a 3D brain template. The significant associations were all positive. The width of the arrows represents the number of functional connections between the regions (see Supplementary Table S2 for the full list of connections). Note, that significant associations were found in the right hemisphere and midline regions. Abbreviations: ACC, anterior cingulate cortex; Hippocampus, hippocampus and parahippocampal gyrus; IFG, inferior frontal gyrus; IPL, inferior parietal lobule; OFC, orbitofrontal cortex; PCC, posterior cingulate cortex; L, left; R, right. *P* < 0.05, FDR corrected for multiple comparisons.

**Figure 2:**
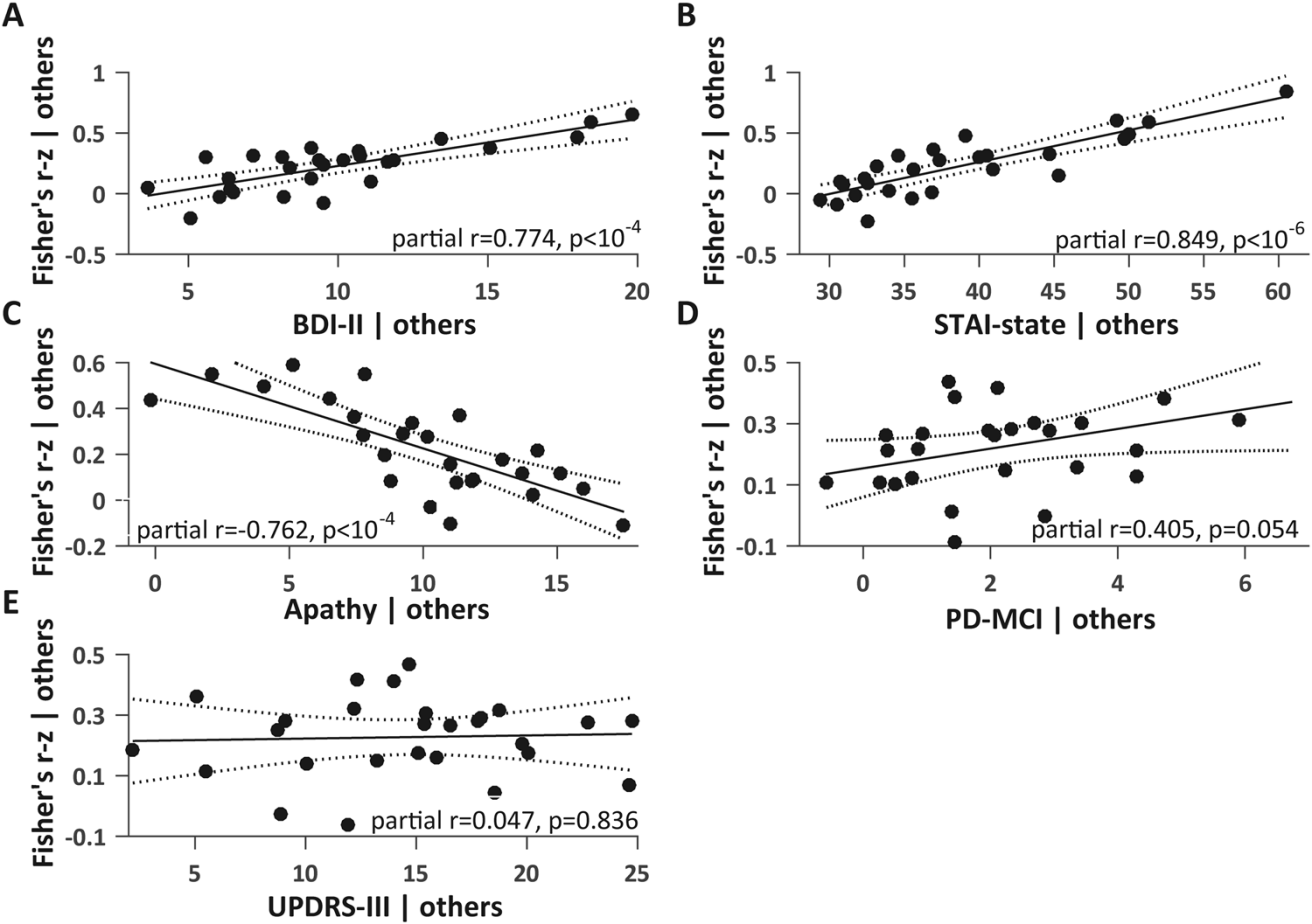
Partial regression plots for a sample ROI-ROI functional connection in PD patients. Partial regression plots are presented to illustrate in PD patients the incremental effect of each neuropsychiatric scale on Fisher’s transformed values by removing the effects of all other scales. The right gyrus rectus - right parahippocampal gyrus functional connection was chosen arbitrarily as an example. The associations are shown between Fisher’s transformed values and **(A)** BDI-II; **(B)** STAI-state; **(C)** apathy; **(D)** PD-MCI and **(E)** UPDRS-III. The y-axis shows the residuals from regressing Fisher’s transformed values against all neuropsychiatric scales other than the scale of interest, and the x-axis shows the residuals from regressing the scale of interest against all other neuropsychiatric scales. The linear fits are shown in solid lines and confidence intervals in dotted lines. Partial r = square root of coefficient of partial determination. The BDI-II, STAI-state and apathy scales are significantly associated with the right gyrus rectus - right parahippocampal gyrus functional connection.

The associations between BDI-II scores and region of interest (ROI)-ROI functional connections (Fisher’s transformed values) in PD patients are shown in Fig. 1 and Supplementary Table S1 (see Fig. 2 for partial regression plots for a sample functional connection). An increase in the severity of depression in PD predicted an increase in the functional connectivity between the bilateral gyrus rectus (part of the orbitofrontal cortex) and the right hippocampus and parahippocampal gyrus, the bilateral anterior cingulate cortex and right supramarginal gyrus, the left caudate and bilateral thalamus, the right inferior frontal gyrus and right posterior cingulate cortex. In particular, associations between BDI-II scores and functional connectivity were only found in the right hemisphere and midline regions.

#### Anxiety in PD and functional connectivity

**Figure 3:**
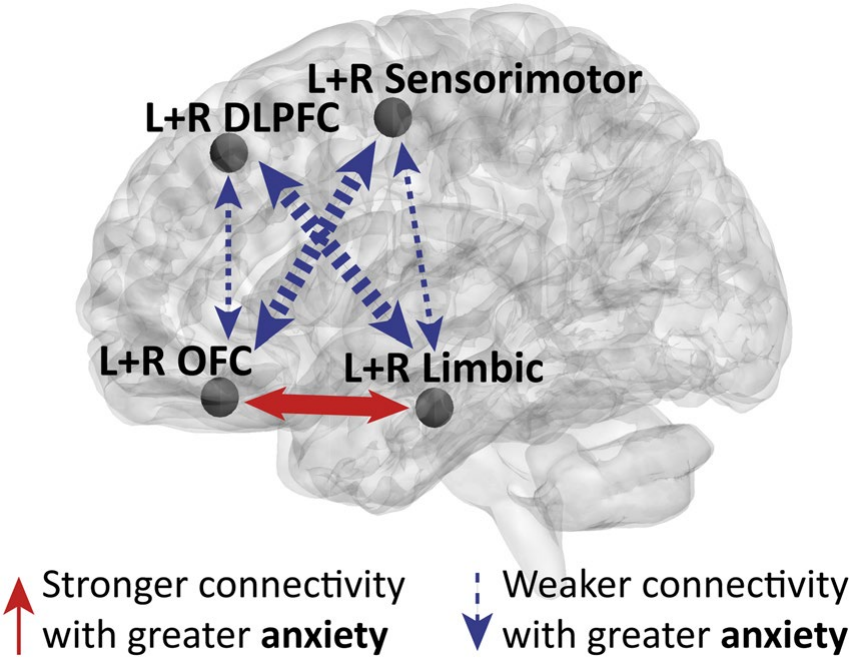
Illustration of functional connections predicted by anxiety in PD patients. Functional connections predicted by anxiety while controlling for other neuropsychiatric scales and UPDRS-III are indicated by arrows on a 3D brain template. Positive associations are indicated by red solid arrows and negative associations by blue dashed arrows. The width of the arrows represents the number of functional connections between the regions (see Supplementary Table S2 for the full list of connections). Abbreviations: DLPFC, dorsolateral prefrontal cortex; Limbic, limbic regions; OFC, orbitofrontal cortex; Sensorimotor, sensorimotor cortex; L, left; R, right. *P* < 0.05, FDR corrected for multiple comparisons.

The associations between STAI-state scores and ROI-ROI functional connections (Fisher’s transformed values) in PD patients are shown in Fig. 3 and Supplementary Table S1. Both positive and negative associations were found. An increase in the severity of anxiety in PD predicted an increase in the functional connectivity between the orbitofrontal cortex (OFC) (including the gyrus rectus) and the amygdala, hippocampus and parahippocampal gyri (42% of positive associations, 16/38). Positive associations with anxiety were also found between the inferior-middle temporal gyri and the OFC, amygdala, hippocampus and parahippocampal gyri (32% of positive associations, 12/38). Note that the majority of positive associations were with connections of the OFC (82% of positive associations, 31/38).

An increase in anxiety predicted a decrease in the functional connections of the sensorimotor cortex: the precentral gyri, postcentral gyri, paracentral lobule and supplementary motor areas (49% of negative associations, 26/53). Most of these connections were with the OFC (62% of sensorimotor cortex connections, 16/26) in addition to connections with the parahippocampal gyri, amygdala and caudate (23% of sensorimotor cortex connections, 6/26). The sensorimotor cortical region with the largest amount of connections was the left precentral gyrus (38% of sensorimotor cortex connections, 10/26). Another pattern of negative associations with anxiety was found for functional connections between the lateral middle-superior frontal gyri and paralimbic-limbic-OFC regions: amygdala, parahippocampal, hippocampus, temporal poles and OFC (36% of negative associations, 19/53). Overall, 45% of all negative associations were with the OFC (24/53).

#### Apathy in PD and functional connectivity

**Figure 4:**
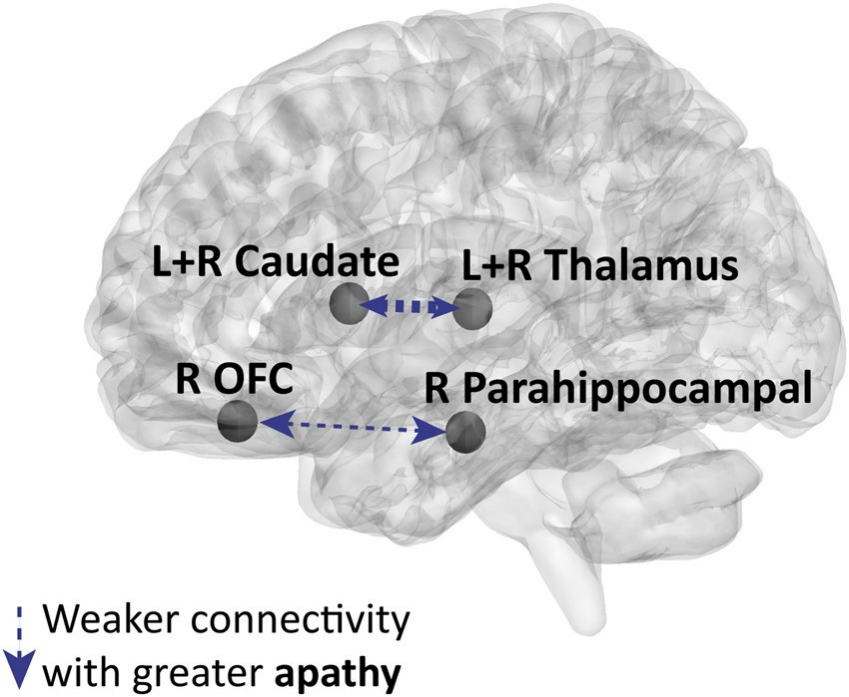
Illustration of functional connections predicted by apathy in PD patients. Functional connections predicted by apathy while controlling for other neuropsychiatric scales and UPDRS-III are indicated by arrows on a 3D brain template. The significant associations were all negative. The width of the arrows represents the number of functional connections between the regions (see Supplementary Table S2 for the full list of connections). Abbreviations: OFC, orbitofrontal cortex; Parahippocampal, parahippocampal gyrus; L, left; R, right. *P* < 0.05, FDR corrected for multiple comparisons.

The associations between Starkstein apathy scores and ROI-ROI functional connections (Fisher’s transformed values) in PD patients are shown in Fig. 4 and Supplementary Table S1. An increase in apathy in PD predicted a decrease in the functional connectivity between the bilateral caudate and bilateral thalamus and between the right gyrus rectus and the right parahippocampal gyrus.

#### Neuropsychiatric symptoms in healthy controls and functional connectivity

The same multiple linear regression model was tested in healthy controls to evaluate whether there are ROI-ROI functional connections (Fisher’s transformed values) that can be predicted by a certain neuropsychiatric symptom. No functional connections were predicted by any of the neuropsychiatric symptoms in healthy controls (see Supplementary Fig. S1 for partial regression plots for a sample functional connection).

#### Differences between PD patients and healthy controls

**Figure 5:**
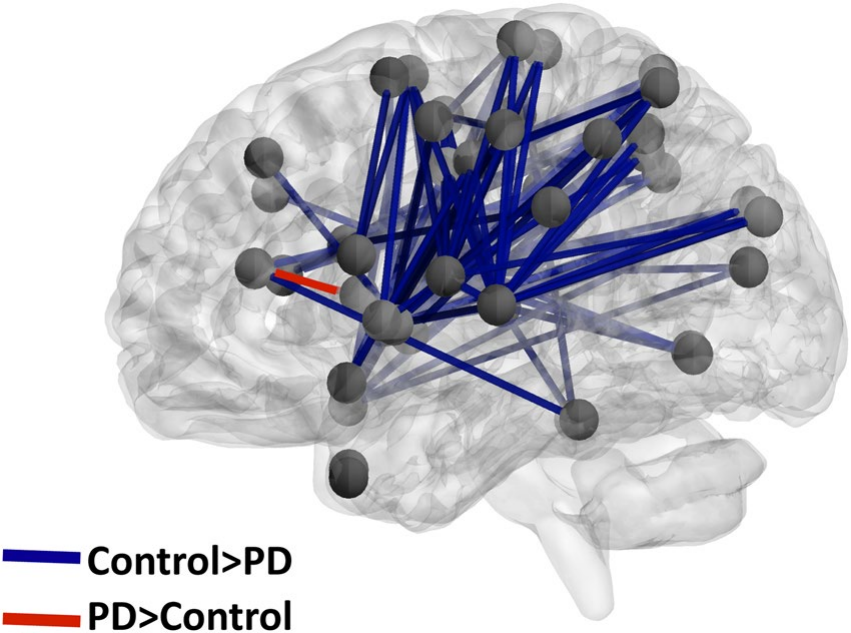
Differences between PD patients and healthy controls in whole-brain functional connectivity. Stronger functional connections in healthy controls are indicated by blue lines and stronger functional connections in PD patients are indicated by red lines (see Supplementary Table S3 for the full list of connections). PD patients showed an extensive reduction in functional connectivity mainly in the sensorimotor, temporal, parietal and subcortical regions. Only the right caudate-right anterior cingulate functional connection was stronger in PD patients (1 out of 122 significant connections). These group-differences were accounted for by either the neuropsychiatric symptoms or the motor deficits of PD patients. Differences between PD patients and healthy controls were evaluated using two sample two-tailed *t* tests with a statistical threshold of *P* < 0.05, FDR corrected for multiple comparisons.

Difference in whole-brain functional connectivity between PD patients and healthy controls were evaluated using two-sample *t* tests with *P* < 0.05, FDR corrected for multiple comparisons and are presented in Fig. 5 and Supplementary Table S2. PD patients showed widespread reduced functional connectivity compared with healthy controls. The right caudate-right anterior cingulate was the only functional connection increased in PD patients. Differences were most noted in functional connections involving the sensorimotor cortex (45% of functional connections, 55/122), cingulate cortex and insula (33%, 40/122), temporal cortex (32%, 39/122), parietal cortex (29%, 35/122) and the basal ganglia (15%, 18/122). Few differences were identified in functional connections of the prefrontal cortex (9%, 11/122). When accounting for differences between PD patients and healthy controls in all neuropsychiatric scales (without accounting for the motor deficits) using a multiple regression model (equation (2) in methods), no between group differences in functional connectivity were identified. Alternatively, when accounting for the motor deficits (equation (3) in methods), no between group differences in functional connectivity were identified.

## Discussion

In the current study, we identified resting-state functional connectivity patterns that vary as a function of a specific neuropsychiatric symptom in PD patients while accounting for the effect of all other neuropsychiatric symptoms. Depression in PD predicted the strength of functional connections of predominant regions previously implicated in depression in PD including the gyrus rectus, parahippocampal gyrus, anterior cingulate cortex and thalamus^9^. While there are quite a few PET and SPECT studies on depression in PD, functional connectivity MRI studies are relatively scarce. Previous functional connectivity studies have found in depressed PD patients an increased functional connectivity of the OFC^10^, inferior frontal gyrus^11^ anterior cingulate^12^ and posterior cingulate^13^, in accordance with our findings, in addition to decreased functional connectivity^12,13^, not identified here. We suggest that this may result from the substantial variability across studies in inclusion and exclusion criteria and statistical approaches. In particular, only one resting-state functional MRI study in depressed PD patients has considered apathy symptoms^14^ and none have accounted for the presence of anxiety. The right hemispheric lateralization found here for the associations between depressive symptoms and functional connectivity is in line with the known hyperactivity of the right hemisphere in idiopathic depression^15^. Claims for the similarity of neural patterns of depression in PD patients and in primary unipolar depression were suggested^16^ yet remain debated^17^.

Anxiety in PD predicted three distinct types of functional connectivity patterns: (i) The first type was positively associated with anxiety and included functional connections between the OFC and temporal-limbic regions: the amygdala, hippocampus, parahippocampal gyri and inferior-middle temporal cortex. (ii) The second type was negatively associated with anxiety and included functional connections between the lateral superior-middle frontal gyri, i.e. the dorsolateral prefrontal cortex (DLPFC), and paralimbic-limbic-OFC regions: the amygdala, hippocampus, parahippocampal gyri, temporal poles and OFC. Note that the paralimbic-limbic-OFC regions involved in the second type are almost identical to the regions of the first type. (iii) The third type was negatively associated with anxiety and included functional connections of the sensorimotor cortex mainly with the OFC.

The few imaging studies conducted on anxiety in PD have mainly applied PET or SPECT techniques^9^. Most of these studies found associations between anxiety and striatal dopamine transporter availability or density^18–20^. No study on anxiety in PD has used functional MRI. Recently, Wang *et al*.^21^, conducted a resting-state FDG-PET study comparing PD patients with and without anxiety. The orbitofrontal cortex was identified as a core region associated with anxiety in PD, in accordance with our findings. The amygdala, hippocampus and OFC (especially its medial part) were all strongly implicated in idiopathic anxiety^22,23^. However, it is less clear how the OFC-limbic functional connections are affected in idiopathic anxiety^24^ with most studies indicating reduced connectivity^25,26^. The finding here of reduced DLPFC-limbic functional connectivity in anxiety is congruent with previous studies in idiopathic anxiety^27^ and suggests diminished top-down regulation of the DLPFC on paralimbic-limbic regions. Note that the OFC and DLPFC are found here to be dissociated in the presence of anxiety. Taken together, the first two types of connectivity patterns propose a limbic-OFC-DLPFC network modulated by anxiety in PD: an increase in anxiety corresponds to stronger OFC-limbic, weaker DLPFC-limbic and weaker DLPFC-OFC connectivity. Considering the emotion regulation model of Phillips *et al*.^28^, these may suggest in anxious PD patients an impaired voluntary emotion regulation of the DLPFC along with an increased automatic emotion regulation of the OFC.

The third type of negative associations between anxiety and the sensorimotor cortex-OFC functional connectivity has not been reported in idiopathic anxiety. We argue that it may be a unique characterization of anxiety in PD and inherently related to its motor dysfunction. There is conflicting evidence as to whether the goal-directed or habitual decision making route is impaired in PD. Loss of dopamine in PD occurs mainly in the sensorimotor part of the striatum which is thought to be responsible for habitual behavior^29^. As a result, it has been hypothesized that there is a primary deficit in PD in habitual control which leads to more reliance on goal-directed control^30^. However, a study directly investigating this hypothesis^31^ showed that habit formation is preserved in PD and found a deficit in goal-directed behavior. The current results may thus reflect the impaired ability of the OFC to guide goal-directed motor actions in anxious PD patients, with a proportional relationship between the level of anxiety and level of impairment in goal-directed behavior. The OFC is known to play a fundamental role in goal-directed behavior^32,33^. In addition, research has shown that stress leads to less goal-directed and more habitual behavior^34,35^. This hypothesis could be further tested experimentally through a task probing goal-directed behavior in PD patients and examining whether a negative association emerges between anxiety and performance of goal-directed behavior and whether the latter is positively associated with sensorimotor-OFC synchrony. Finally, it is noteworthy that most functional connections of the sensorimotor cortex, OFC and DLPFC in anxiety were from these regions at the left hemisphere (69%, 73% and 74% respectively). This left hemisphere lateralization was not found for connections of the temporal, paralimbic or limbic regions (only 49% of these regions were at the left hemisphere).

The findings here of negative associations between apathy in PD and functional connectivity of the gyrus rectus (part of the OFC), caudate and thalamus are congruent with the view that apathy may reflect a dysfunction in the prefrontal cortex-basal ganglia circuits^36,37^. The only functional connectivity MRI study to date on apathy in PD has found negative correlations between fronto-striatal functional connections and apathy while controlling for depression and cognitive impairment^38^ (but not controlling for anxiety), in accordance with our findings. The majority of studies on apathy in PD have been PET and SPECT studies which mainly found inverse associations between apathy in PD and cerebral metabolism in the striatum, prefrontal and limbic regions^39–41^ but positive associations were also found^39^.

Several functional connections were found to be uniquely associated with more than one neuropsychiatric scale. These functional connections included the bilateral gyrus rectus-right parahippocampal gyri, right gyrus rectus-right hippocampus, left caudate-bilateral thalamus, and left orbitofrontal gyrus-left frontal medial orbital cortex. Remarkably, anxiety and depression had opposite effects compared with apathy on the right gyrus rectus-right parahippocampal gyrus and the left caudate-bilateral thalamus functional connections. While the strength of these connections increased with higher levels of anxiety or depression, it decreased with higher levels of apathy. This highlights the importance of considering simultaneously the impact of all neuropsychiatric symptoms on brain measures.

Compared with healthy controls, PD patients showed an extensive reduction in functional connectivity mainly in the sensorimotor, temporal, parietal and subcortical regions. These differences were accounted for by either the neuropsychiatric symptoms or the motor deficits of PD patients. Decreased functional connectivity in PD patients relative to healthy controls has been previously demonstrated for drug naïve PD patients in limbic and motor regions^42^ and for patients ON dopaminergic medication in the temporal and parietal cortices^43^ and in the cortical and subcortical motor system^44,45^.

## Limitations

PD patients were tested ON dopaminergic medication and possible pharmacological effects on functional connectivity measures cannot be ruled out. However, some PD patients suffer from mood symptoms predominantly during their OFF medication state^46^. Therefore, assessment of patients ON medication was preferred. The relatively modest sample size may limit the generalizability of the findings and studies with greater number of patients are needed to strengthen the results. The lack of testing for unique functional networks associated with cognitive impairment is a limitation. Due to different domains composing the cognitive assessment and great variability within these domains in PD patients, a cognitive summary metric was considered general and not appropriate to identify meaningful neural functional networks. Thus, the cognitive summary score was not used to predict functional networks and only included as a covariate to control for overall differences in cognitive status. In addition, the range of neuropsychiatric symptoms in our sample of patients was wider for anxiety and included some individuals with more severe anxious symptoms compared to the range of other symptoms. Finally, future studies comparing anxious PD patients with a matched group of idiopathic anxious subjects are needed and may further shed light on the specific neural mechanisms of anxiety in PD.

## Conclusions

Anxiety in PD predicted three types of functional connectivity patterns not described before: (i) increased limbic-OFC; (ii) decreased DLPFC-limbic and DLPFC-OFC and (iii) decreased sensorimotor cortex-OFC. The first two types suggest less voluntary and more automatic emotion regulation in anxious PD patients. The diminished sensorimotor-OFC connectivity is argued to be specific to PD and reflect an impaired ability of the OFC to guide goal-directed motor actions in anxious PD patients. Widespread reduced functional connectivity was identified in PD patients compared with healthy controls and explained by either the neuropsychiatric or motor symptoms. This study provides further insights into the separate mechanisms involved in neuropsychiatric symptoms of PD and particularly those of anxiety.

## Methods

### Patients

The sample was composed of 37 patients (16 women, age: 64.6 ± 7.51 years) with a diagnosis of idiopathic PD based on the United Kingdom Parkinson’s disease Society Brain Bank Diagnostic Criteria for PD^47^. All patients were in the intermediate phase associated with motor complications^48^. Inclusion criteria were: treatment with levodopa in monotherapy or in combination with a dopamine agonist, stable medication for the previous four weeks and Hoehn and Yahr stage below III while on dopaminergic medication. Exclusion criteria were: a history of psychotic symptoms, treatment with antipsychotics, treatment with deep brain stimulation, dementia or any concomitant disease or condition compromising the cognitive state. Ten patients were not included in the final group of subjects due to a change in diagnosis (one subject with multiple system atrophy), missing neuropsychiatric data, severe vascular lesions, atrophy or any single time point of head motion >3 mm or 3° during the MRI scan, which yielded a final sample of 27 patients (12 women, age: 64.9 ± 7.9 years). All patients were right handed according to the Edinburgh Handedness Inventory. Patients were evaluated on their regular dopaminergic medication (“ON” condition) and their motor function and disease severity were assessed using the Unified Parkinson’s Disease Rating Scale motor examination (UPDRS-III)^49^ and the Hoehn and Yahr rating scale. In addition, a matched group of 41 healthy controls (22 women, age: 63.9 ± 8.1 years) was recruited. Twelve control subjects were not included in the final group of subjects due to the presence of moderate neuropsychiatric symptoms, severe vascular lesions or atrophy which yielded a final sample of 29 control subjects (14 women, age: 63.3 ± 8 years). This study was approved by the Ethical Committee of the General University Hospital in Prague, Czech Republic. All participants provided written informed consent prior to inclusion in the study in compliance with the Declaration of Helsinki and all methods were performed in accordance with the relevant guidelines and regulations.

### Clinical evaluation

All study subjects, PD patients and controls, underwent clinical evaluations by a neurologist and a neuropsychologist prior to inclusion in the study. Depression was measured using the Beck Depression Inventory, second edition (BDI-II)^50^. Anxiety was measured using the Spielberger State-Trait Anxiety Inventory (STAI)^51^. Apathy was assessed on the Starkstein Apathy Scale (AS)^52^ as recommended in PD neuropsychiatric research^53^. Montreal Cognitive Assessment (MoCA)^54^ was administered followed by a neuropsychological battery to measure cognitive function in PD as recommended by Litvan *et al*.^55,56^ (level II- comprehensive assessment, see supplementary methods for the full list of tests used). In the comprehensive assessment two neurophysiological tests were used for each of the following five domains: attention and working memory, executive function, language, short term memory and visuospatial function. The results of the neuropsychological evaluation as a function of cognitive domain are summarized in Supplementary Table S3. The score on each test was transformed into a z-score using the Rankit formula^57^. Patients who scored >1.5 standard deviations below the average z-score derived from the group of matched healthy controls were considered impaired on the test and a “PD-MCI” composite score was computed by the linear sum of the number of deficits on the ten neurophysiological tests. The PD-MCI score was used in the regression analysis as a covariate to control for variance in cognitive impairment. The main demographic and clinical characteristics of the subjects are summarized in Table 1. Cognitive assessment and completion of BDI-II and apathy scales were carried out during a preliminary visit approximately two weeks before the MRI scan. UPDRS-III and STAI were evaluated immediately before the MRI scan, to account for state symptoms and confirm the ON condition for PD patients.

**Table 1:** Demographic and clinical characteristics of PD patients and healthy control subjects.

Demographic and clinical variables	PD patients, Mean ± SD (range)	Healthy controls, Mean ± SD (range)
Age, years	64.9 ± 7.9 (46–82)	63.3 ± 8 (46–83)
Gender (male/female)	15/12	15/14
Education, years	13.5 ± 2.7 (8–18)	14.7 ± 3.5 (11–25)
Disease duration, years	11.1 ± 3.7 (4–18)	—
H/Y stage ON medication	2 ± 0.5 (1–3)	—
UPDRS-III ON medication	14.4 ± 7.1 (4–31)	—
LEDD	1306.1 ± 616.7 (450–2371)	—
BDI-II	10 ± 4.8 (2–21)	6.9 ± 5.2 (0–19)
STAI-X1 (state)	38.7 ± 9.4 (28–64)	32.7 ± 5.7 (20–47)
STAI-X2 (trait)	41.8 ± 8.9 (27–56)	35.1 ± 8.2 (22–55)
Starkstein apathy scale	10 ± 4.7 (0–19)	9.58 ± 4.4 (1–21)
MoCA	26 ± 2.2 (20–29)	26.6 ± 2.2 (22–30)
PD-MCI score	2 ± 2.1 (0–7)	0.14 ± 0.3 (0–1)

Abbreviations: H/Y, Hoehn and Yahr; LEDD, Levodopa equivalent daily dose^65^; BDI-II, Beck Depression Inventory, second edition; STAI, Spielberger State-Trait Anxiety Inventory; MoCA, Montreal Cognitive Assessment.

### MRI data acquisition

Magnetic resonance images were acquired with a 3T MR scanner (Magnetom Skyra, Siemens, Germany). Patients were evaluated on their regular dopaminergic medication. Each participant underwent 10-minute resting-state functional MRI during which they were instructed to fixate on a visual crosshair, remain still and awake. Wakefulness was monitored during the whole scan using an MRI compatible camera. Functional images were acquired using T_2_^*^-weighted gradient-echo echo-planar imaging (GE-EPI) sequence with TR = 2 sec, TE = 30 ms, image matrix = 64 × 64, field of view = 192 × 192 mm, flip angle = 90°, resolution = 3 × 3 × 3 mm, interslice gap = 0.45 mm. Each brain volume comprised 30 axial slices, and each functional run contained 300 image volumes. High resolution anatomical images were acquired using a sagittal T1-weighted MP-RAGE sequence with TR = 2.2 sec, TE = 2.43 ms, resolution = 1 × 1 × 1 mm; and a T2-weighted 2D sequence with TR = 3.2 sec, TE = 9 ms, resolution = 0.9 × 0.9 × 3 mm. T1-weighted images were acquired for coregistration and normalization of the functional images and T2-weighted images were acquired for diagnostic purpose to rule out significant atrophy or any other pathological brain changes.

### Functional MRI data preprocessing and functional connectivity analysis

Standard initial preprocessing of functional MRI data used Statistical Parametric Mapping (SPM8, Wellcome Trust Centre for Neuroimaging, London, United Kingdom, http://www.fil.ion.ucl.ac.uk/spm/software/spm8 *)*. First, functional images were spatially realigned, coregistered to high resolution T1 anatomical images, normalized to Montreal Neurological Institute space and resampled at an isotropic voxel size of 2 mm. The normalized images were smoothed with an isotropic 8 mm full-width-at-half-maximum Gaussian kernel. Functional MRI data were further preprocessed using CONN toolbox^58^. The six motion parameters were removed by regression and despiking was applied. Furthermore, the aCompCor method^59^ was applied to regress out the first principal component of the CSF and white matter signals. This was done to minimize the effects of potential physiological and non-neuronal signals such as cardiac and respiratory signals, without the risk of artificially introducing anticorrelations into the functional connectivity estimates. Specifically, the principal components of the CSF and white matter were chosen and not their average signals, as the aCompCor was shown to remove motion artifacts more effectively along with preserving signals of interest better than other nuisance removal strategies^60,61^. In the next steps, linear detrending and band-pass filtering (0.01–0.1 Hz) were applied.

Region of interest (ROI)-ROI functional connectivity analysis was done using 84 cerebral Automated Anatomical Labeling (AAL)^62^ regions as ROIs. Primary visual and auditory regions were excluded from analysis; i.e. the bilateral cuneus, calcarine cortex and Heschl’s gyri, since they were hypothesized not to be involved in the neuropsychiatric symptoms. The primary sensorimotor and motor cortices were included in the analysis since they are implicated in the pathophysiology of PD and they were posited to show associations with neuropsychiatric symptoms (for the same analysis when including all 90 AAL regions, see Supplementary Table S4. This analysis did not introduce any qualitative changes in the findings for any of the neuropsychiatric symptoms). Pearson’s correlations were computed between each pair of ROIs and Fisher’s transform was applied to the correlation values. BrainNet Viewer^63^ was used for visualization of the results.

### Statistical analysis

A multiple linear regression model was tested in CONN to evaluate the unique ROI-ROI functional connections (Fisher’s transformed values) predicted by each neuropsychiatric symptom in PD patients while statistically controlling for the contribution of all other neuropsychiatric scales and the motor deficits. The following regression model was used:

1 $${{\rm{FC}}}_{{\rm{j}}}={{\rm{\beta }}}_{{\rm{j}},0}+{{\rm{\beta }}}_{{\rm{j}},1}\cdot {{\rm{X}}}_{1}+{{\rm{\beta }}}_{{\rm{j}},2}\cdot {{\rm{X}}}_{2}+{{\rm{\beta }}}_{{\rm{j}},3}\cdot {{\rm{X}}}_{3}+{{\rm{\beta }}}_{{\rm{j}},4}\cdot {{\rm{X}}}_{4}+{{\rm{\beta }}}_{{\rm{j}},5}\cdot {{\rm{X}}}_{5}+{{\rm{\varepsilon }}}_{{\rm{j}}}$$

where FC_j_ is the vector of Fisher’s transformed values for functional connection ‘j’ between a pair of AAL regions across subjects, ${X}_{i=1,\ldots ,5}\,\,$are the BDI-II, STAI-state, apathy, PD-MCI and UPDRS-III scores, β_ji_′s are the regression coefficients for functional connection ‘j’ and ε_j_ is the error term. PD-MCI and UPDRS-III were included in the model as covariates of no interest, to account for variance in cognitive impairment and motor function, respectively, among PD patients. STAI-trait was not included in the regression model due to its high correlation with STAI-state (r = 0.778, *P* < 0.00001). A least-squares method was applied to fit the model to the data. One sample two-tailed *t* tests with a statistical threshold of *P* < 0.05, FDR corrected for multiple comparisons were used^64^.

Differences in whole-brain functional connectivity between PD patients and healthy controls were evaluated between the AAL ROIs using two sample two-tailed *t* tests with a statistical threshold of *P* < 0.05, FDR corrected for multiple comparisons. To further examine the basis of these between group differences, two different multiple regression models were tested:

2 $${{\rm{FC}}}_{{\rm{j}}}={{\rm{\beta }}}_{{\rm{j}},0}+{{\rm{\beta }}}_{{\rm{j}},1}\cdot {{\rm{X}}}_{{\rm{group}}}+{{\rm{\beta }}}_{{\rm{j}},2}\cdot {{\rm{X}}}_{2}+{{\rm{\beta }}}_{{\rm{j}},3}\cdot {{\rm{X}}}_{3}+{{\rm{\beta }}}_{{\rm{j}},4}\cdot {{\rm{X}}}_{4}+{{\rm{\beta }}}_{{\rm{j}},5}\cdot {{\rm{X}}}_{5}+{{\rm{\varepsilon }}}_{{\rm{j}}}$$

where FC_j_ is the vector of Fisher’s transformed values for functional connection ‘j’, X_group_ is a categorical variable encoding the two groups of subjects, ${{\rm{X}}}_{2},{{\rm{X}}}_{3},{{\rm{X}}}_{4},{{\rm{X}}}_{5}$ are the BDI-II, anxiety, apathy and PD-MCI scores for both groups, β_ji_′s are the regression coefficients for functional connection ‘j’ and ε_j_ is the error term. This model was chosen to examine whether the differences between PD patients and healthy controls can be explained by the neuropsychiatric symptoms. In addition, an alternative multiple regression model was tested:

3 $${{\rm{FC}}}_{{\rm{j}}}={{\rm{\beta }}}_{{\rm{j}}0}+{{\rm{\beta }}}_{{\rm{j}}1}\cdot {{\rm{X}}}_{{\rm{group}}}+{{\rm{\beta }}}_{{\rm{j}}2}\cdot {{\rm{X}}}_{2}+{{\rm{\varepsilon }}}_{{\rm{j}}}$$

where FC_j_ is the vector of Fisher’s transformed values for functional connection ‘j’, X_group_ is a categorical variable encoding the two groups of subjects, X_2_ is a vector of the UPDRS-III scores, β_ji_′s are the regression coefficients for functional connection ‘j’ and ε_j_ is the error term. This model was chosen to examine whether the differences between PD patients and healthy controls can be explained by the motor symptoms of PD patients.

### Data availability

The datasets analyzed during the current study are available from the corresponding author on reasonable request.

## Electronic supplementary material

Supplementary information

## References

[1] Castrioto A, Thobois S, Carnicella S, Maillet A, Krack P (2016). Emotional manifestations of PD: Neurobiological basis. Mov. Disord..

[2] Barone P (2009). The PRIAMO study: A multicenter assessment of nonmotor symptoms and their impact on quality of life in Parkinson’s disease. Mov. Disord..

[3] Langston JW (2006). The Parkinson’s complex: parkinsonism is just the tip of the iceberg. Ann. Neurol..

[4] Chaudhuri KR (2006). International multicenter pilot study of the first comprehensive self-completed nonmotor symptoms questionnaire for Parkinson’s disease: the NMSQuest study. Mov. Disord..

[5] Nègre-Pagès L (2010). Anxious and depressive symptoms in Parkinson’s disease: the French cross-sectionnal DoPaMiP study. Mov. Disord..

[6] Aarsland D (2007). Neuropsychiatric symptoms in patients with Parkinson’s disease and dementia: frequency, profile and associated care giver stress. J. Neurol. Neurosurg. Psychiatry.

[7] Dissanayaka NNW (2010). Anxiety disorders in Parkinson’s disease: prevalence and risk factors. Mov. Disord..

[8] Starkstein SE (2009). The syndromal validity and nosological position of apathy in Parkinson’s disease. Mov. Disord..

[9] Wen M-C, Chan LL, Tan LCS, Tan EK (2016). Depression, anxiety, and apathy in Parkinson’s disease: insights from neuroimaging studies. Eur. J. Neurol..

[10] Luo C (2014). Resting-state fMRI study on drug-naive patients with Parkinson’s disease and with depression. J. Neurol. Neurosurg. Psychiatry.

[11] Sheng K (2014). Altered Spontaneous Brain Activity in Patients with Parkinson’s Disease Accompanied by Depressive Symptoms, as Revealed by Regional Homogeneity and Functional Connectivity in the Prefrontal-Limbic System. PLoS One.

[12] Wei L (2017). Aberrant Intra- and Internetwork Functional Connectivity in Depressed Parkinson’s Disease. Sci. Rep..

[13] Lou Y (2015). Altered brain network centrality in depressed Parkinson’s disease patients. Mov. Disord..

[14] Skidmore FM (2013). Apathy, depression, and motor symptoms have distinct and separable resting activity patterns in idiopathic Parkinson disease. Neuroimage.

[15] Hecht D (2010). Depression and the hyperactive right-hemisphere. Neurosci. Res..

[16] Mayberg HS (2003). Modulating dysfunctional limbic-cortical circuits in depression: towards development of brain-based algorithms for diagnosis and optimised treatment. Br. Med. Bull..

[17] Liang P (2016). Altered directional connectivity between emotion network and motor network in Parkinson’s disease with depression. Medicine..

[18] Erro R (2012). Anxiety is associated with striatal dopamine transporter availability in newly diagnosed untreated Parkinson’s disease patients. Parkinsonism Relat. Disord..

[19] Moriyama TS (2011). Increased dopamine transporter density in Parkinson’s disease patients with Social Anxiety Disorder. J. Neurol. Sci..

[20] Weintraub D (2005). Striatal dopamine transporter imaging correlates with anxiety and depression symptoms in Parkinson’s disease. J. Nucl. Med..

[21] Schapira AH, Ray Chaudhuri K, Jenner P (2017). Non-motor features of Parkinson disease. Nat. Rev. Neurosci..

[22] Shin LM, Liberzon I (2010). The Neurocircuitry of Fear, Stress, and Anxiety Disorders. Neuropsychopharmacology.

[23] Taylor JM, Whalen PJ (2015). Neuroimaging and Anxiety: the Neural Substrates of Pathological and Non-pathological Anxiety. Curr. Psychiatry Rep..

[24] Sylvester CM (2012). Functional network dysfunction in anxiety and anxiety disorders. Trends Neurosci..

[25] Kim MJ, Gee DG, Loucks RA, Davis FC, Whalen PJ (2011). Anxiety Dissociates Dorsal and Ventral Medial Prefrontal Cortex Functional Connectivity with the Amygdala at Rest. Cereb. Cortex.

[26] Hahn A (2011). Reduced resting-state functional connectivity between amygdala and orbitofrontal cortex in social anxiety disorder. Neuroimage.

[27] Etkin A, Prater KE, Schatzberg AF, Menon V, Greicius MD (2009). Disrupted Amygdalar Subregion Functional Connectivity and Evidence of a Compensatory Network in Generalized Anxiety Disorder. Arch. Gen. Psychiatry.

[28] Phillips ML, Ladouceur CD, Drevets WC (2008). A neural model of voluntary and automatic emotion regulation: implications for understanding the pathophysiology and neurodevelopment of bipolar disorder. Mol. Psychiatry.

[29] Balleine BW, O’Doherty JP (2010). Human and rodent homologies in action control: corticostriatal determinants of goal-directed and habitual action. Neuropsychopharmacology.

[30] Redgrave P (2010). Goal-directed and habitual control in the basal ganglia: implications for Parkinson’s disease. Nat. Rev. Neurosci..

[31] de Wit S, Barker RA, Dickinson AD, Cools R (2011). Habitual versus goal-directed action control in Parkinson disease. J. Cogn. Neurosci..

[32] FitzGerald TH, Friston KJ, Dolan RJ (2012). Action-specific value signals in reward-related regions of the human brain. J. Neurosci..

[33] Schoenbaum G, Roesch M (2005). Orbitofrontal cortex, associative learning, and expectancies. Neuron.

[34] Dias-Ferreira E (2009). Chronic stress causes frontostriatal reorganization and affects decision-making. Science.

[35] Schwabe L, Wolf OT (2011). Stress-induced modulation of instrumental behavior: from goal-directed to habitual control of action. Behav. Brain Res..

[36] Levy R, Dubois B (2006). Apathy and the functional anatomy of the prefrontal cortex-basal ganglia circuits. Cereb. Cortex.

[37] Wang X (2017). Cerebral metabolic change in Parkinson’s disease patients with anxiety: A FDG-PET study. Neurosci. Lett..

[38] Baggio HC (2015). Resting-state frontostriatal functional connectivity in Parkinson’s disease-related apathy. Mov. Disord..

[39] Le Jeune F (2009). Subthalamic nucleus stimulation in Parkinson disease induces apathy: a PET study. Neurology.

[40] Santangelo G (2015). Apathy and striatal dopamine transporter levels in de-novo, untreated Parkinson’s disease patients. Parkinsonism Relat. Disord..

[41] Robert GH (2014). Preoperative factors of apathy in subthalamic stimulated Parkinson disease: a PET study. Neurology.

[42] Luo C (2014). Reduced functional connectivity in early-stage drug-naive Parkinson’s disease: a resting-state fMRI study. Neurobiol. Aging.

[43] Tessitore A (2012). Default-mode network connectivity in cognitively unimpaired patients with Parkinson disease. Neurology.

[44] Hacker CD, Perlmutter JS, Criswell SR, Ances BM, Snyder AZ (2012). Resting state functional connectivity of the striatum in Parkinson’s disease. Brain.

[45] New AB (2015). The intrinsic resting state voice network in Parkinson’s disease. Hum. Brain Mapp..

[46] Witjas T (2002). Nonmotor fluctuations in Parkinson’s disease: frequent and disabling. Neurology.

[47] Hughes AJ, Daniel SE, Kilford L, Lees AJ (1992). Accuracy of clinical diagnosis of idiopathic Parkinson’s disease: a clinico-pathological study of 100 cases. J. Neurol. Neurosurg. Psychiatry.

[48] Deuschl G (2013). Stimulation of the subthalamic nucleus at an earlier disease stage of Parkinson’s disease: Concept and standards of the EARLYSTIM-study. Park. Relat. Disord..

[49] Fahn, S. & Elton, R. In *Recent developments in* Parkinson’s Disease (eds Fahn, S., Marsden, C., Calne, D. & Goldstein, M.) 153–63 (FlorhamPark, NJ: MacMillanHealthCare, 1987).

[50] Beck AT, Steer RA, Brown GK (1996). Beck depression inventory-II. San Antonio.

[51] Spielberger, C. D., Gorsuch, R. L. & Lushene, R. E. Manual for the State-Trait Anxiety Inventory. *Palo Alto* (1970).

[52] Starkstein SE (1992). Reliability, validity, and clinical correlates of apathy in Parkinson’s disease. J. Neuropsychiatry Clin. Neurosci..

[53] Leentjens AF (2008). Apathy and anhedonia rating scales in Parkinson’s disease: Critique and recommendations. Mov. Disord..

[54] Nasreddine ZS (2005). The Montreal Cognitive Assessment, MoCA: a brief screening tool for mild cognitive impairment. J. Am. Geriatr. Soc..

[55] Litvan I (2012). Diagnostic criteria for mild cognitive impairment in Parkinson’s disease: Movement Disorder Society Task Force guidelines. Mov. Disord..

[56] Bezdicek, O. *et al*. The Diagnostic Accuracy of Parkinson’s Disease Mild Cognitive Impairment Battery Using the Movement Disorder Society Task Force Criteria. *Mov. Disord. Clin. Pract*. (2016). 10.1002/mdc3.12391 PMC6174468 30363396

[57] Solomon SR, Sawilowsky SS (2009). Impact of Rank-Based Normalizing Transformations on the Accuracy of Test Scores. J. Mod. Appl. Stat. Methods.

[58] Whitfield-Gabrieli S, Nieto-Castanon A (2012). Conn: a functional connectivity toolbox for correlated and anticorrelated brain networks. Brain Connect..

[59] Behzadi Y, Restom K, Liau J, Liu TT (2007). A component based noise correction method (CompCor) for BOLD and perfusion based fMRI. Neuroimage.

[60] Muschelli J (2014). Reduction of motion-related artifacts in resting state fMRI using aCompCor. Neuroimage.

[61] Chai XJ, Castañón AN, Öngür D, Whitfield-Gabrieli S (2012). Anticorrelations in resting state networks without global signal regression. Neuroimage.

[62] Tzourio-Mazoyer N (2002). Automated anatomical labeling of activations in SPM using a macroscopic anatomical parcellation of the MNI MRI single-subject brain. Neuroimage.

[63] Xia M, Wang J, He Y (2013). BrainNet Viewer: a network visualization tool for human brain connectomics. PLoS One.

[64] Yekutieli D, Benjamini Y (1999). Resampling-based false discovery rate controlling multiple test procedures for correlated test statistics. J. Stat. Plan. Inference.

[65] Tomlinson CL (2010). Systematic review of levodopa dose equivalency reporting in Parkinson's disease. Mov. Disord..

