# Supplementary material for: Separate neural representations of depression, anxiety and apathy in Parkinson’s disease
Source: Sci Rep. 2017 Sep 22;7:12164. doi: 10.1038/s41598-017-12457-6 (PMC5610322; doi:10.1038/s41598-017-12457-6)
Supplement: Supplementary file 1 — Supplementary information [file 41598_2017_12457_MOESM1_ESM.pdf]

Supplementary information

Supplement to:

Separate neural representations of depression, anxiety and apathy in Parkinson's disease

Rotem Dan, Filip Růžička, Ondrej Bezdicek, Evžen Růžička, Jan Roth, Josef Vymazal, Gadi Goelman and Robert Jech

List of contents

- Supplementary Table S1. Functional connections predicted by depression, anxiety and apathy in PD patients.
- Supplementary Figure S1. Partial regression plots for a sample ROI-ROI functional connection in healthy controls.
- Supplementary Table S2. Differences between PD patients and healthy controls in whole-brain functional connectivity.
- Supplementary Methods. Neuropsychological battery used to assess cognitive function of Parkinson’s disease patients.
- Supplementary Table S3. Results of the neuropsychological evaluation as a function of cognitive domain.
- Supplementary Table S4. Functional connections predicted by depression, anxiety and apathy in PD patients. Same analysis as in Supplementary Table S1 with the primary auditory and visual regions included.

Supplementary Table S1. Functional connections predicted by depression, anxiety and apathy in PD patients.

ROI-ROI functional connection	*t* statistic (df=21)	*P* value (FDR corrected)[a]
**Depression**		
R gyrus rectus – R parahippocampal gyrus	5.6	0.0458
R anterior cingulate – R supramarginal gyrus	5.18	0.0458
L caudate – R thalamus	5.18	0.0458
R inferior frontal gyrus, opercular part – R angular gyrus	5.01	0.0482
L caudate – L thalamus	4.72	0.0482
L gyrus rectus – R parahippocampal gyrus	4.71	0.0482
R gyrus rectus – R hippocampus	4.7	0.0482
L anterior cingulate – R supramarginal gyrus	4.69	0.0482
R inferior frontal gyrus, triangular part – R posterior cingulate	4.69	0.0482
**Anxiety**		
Positive associations		
L gyrus rectus – R parahippocampal gyrus	8.64	0.0001
R gyrus rectus – R parahippocampal gyrus	7.36	0.0005
L gyrus rectus – L parahippocampal gyrus	5.38	0.0147
L subcallosal area – L superior frontal gyrus, medial orbital part	5.35	0.0147
R gyrus rectus – L superior frontal gyrus, orbital part	5.3	0.0147
L amygdala – R middle temporal gyrus	5.24	0.0147
R gyrus rectus – L parahippocampal gyrus	5.18	0.0147
L hippocampus – R middle temporal gyrus	5.12	0.0147
L gyrus rectus – R subcallosal area	5.07	0.0147
R gyrus rectus – L inferior temporal gyrus	4.91	0.0155
R amygdala – R middle temporal gyrus	4.9	0.0155
L gyrus rectus – L inferior temporal gyrus	4.79	0.0191
L gyrus rectus – R hippocampus	4.67	0.0223
L gyrus rectus – R amygdala	4.64	0.0223
L parahippocampal gyrus – L inferior frontal gyrus, orbital part	4.56	0.0233
R hippocampus – R middle temporal gyrus	4.55	0.0233
L olfactory cortex – L superior frontal gyrus, orbital part	4.52	0.0233
L olfactory cortex – L gyrus rectus	4.52	0.0233
L olfactory cortex – R superior frontal gyrus, medial orbital part	4.47	0.0239
L hippocampus – L superior frontal gyrus, orbital part	4.39	0.0263
L inferior temporal gyrus – L superior frontal gyrus, orbital part	4.37	0.0263
L hippocampus – L gyrus rectus	4.35	0.0266
R olfactory cortex – R gyrus rectus	4.23	0.03
R amygdala – R gyrus rectus	4.19	0.0311
L parahippocampal gyrus – L superior frontal gyrus, orbital part	4.17	0.0311
L gyrus rectus – L superior frontal gyrus, orbital part	4.08	0.0354
L gyrus rectus – R gyrus rectus	4.02	0.0389
R parahippocampal gyrus – R middle temporal gyrus	3.95	0.0418
R gyrus rectus – R middle temporal gyrus	3.94	0.0418
R amygdala – R middle temporal gyrus	3.92	0.0418
L parahippocampal gyrus – L middle frontal gyrus, orbital part	3.91	0.0418
R inferior temporal gyrus – L superior frontal gyrus, orbital part	3.9	0.0418
R parahippocampal gyrus – L inferior frontal gyrus, orbital part	3.84	0.0441

R gyrus rectus – R inferior temporal gyrus	3.84	0.0441
L insula – R lingual gyrus	3.81	0.0453
R gyrus rectus – R hippocampus	3.81	0.0453
R gyrus rectus – L amygdala	3.8	0.0454
L amygdala – L middle frontal gyrus, orbital part	3.73	0.0481
Negative associations		
L precentral gyrus – L superior frontal gyrus, orbital part	-5.48	0.0147
R amygdala – L superior frontal gyrus, dorsolateral	-5.18	0.0147
R precentral gyrus – L superior frontal gyrus, orbital part	-5.03	0.0147
L precentral gyrus – L inferior frontal gyrus, orbital part	-5.02	0.0147
L superior frontal gyrus, dorsolateral – L inferior frontal gyrus, orbital part	-5.01	0.0147
L postcentral gyrus – L middle frontal gyrus, orbital part	-4.94	0.0155
L precentral gyrus – L middle frontal gyrus, orbital part	-4.76	0.0192
R amygdala – L middle frontal gyrus	-4.66	0.0223
L rolandic operculum – R inferior occipital gyrus	-4.61	0.0228
L inferior frontal gyrus, orbital part – L middle frontal gyrus	-4.57	0.0233
R parahippocampal gyrus – L middle frontal gyrus	-4.5	0.0233
L postcentral gyrus – L superior frontal gyrus, orbital part	-4.49	0.0233
R superior frontal gyrus, dorsolateral – L temporal pole, middle temporal gyrus	-4.42	0.0261
L superior frontal gyrus, dorsolateral – L temporal pole, superior temporal gyrus	-4.38	0.0263
R superior frontal gyrus, dorsolateral – R amygdala	-4.37	0.0263
R supplementary motor area – R superior frontal gyrus, medial	-4.34	0.0266
L supplementary motor area – L superior frontal gyrus, orbital part	-4.29	0.0284
R supplementary motor area – L caudate	-4.29	0.0284
R middle frontal gyrus – R insula	-4.28	0.0284
L superior frontal gyrus, dorsolateral – L amygdala	-4.26	0.0289
L middle frontal gyrus – R hippocampus	-4.23	0.03
R supplementary motor area – L superior frontal gyrus, orbital part	-4.2	0.0311
L precentral gyrus – L gyrus rectus	-4.17	0.0311
L precentral gyrus – L amygdala	-4.17	0.0311
L precentral gyrus – R inferior frontal gyrus, orbital part	-4.12	0.034
L gyrus rectus – L supramarginal gyrus	-4.11	0.0345
L middle frontal gyrus – L superior frontal gyrus, medial orbital part	-4.09	0.035
R precentral gyrus – R superior frontal gyrus, orbital part	-4.04	0.0384
L superior frontal gyrus, orbital part – R inferior frontal gyrus, opercular part	-4.02	0.0389
R superior frontal gyrus, dorsolateral – L middle temporal gyrus	-3.98	0.0415
L supplementary motor area – L caudate	-3.95	0.0418
R paracentral lobule – R superior frontal gyrus, medial part	-3.94	0.0418
R inferior frontal gyrus, orbital part – L supramarginal gyrus	-3.94	0.0418
L postcentral gyrus – L parahippocampal gyrus	-3.93	0.0418
L precentral gyrus – R parahippocampal gyrus	-3.92	0.0418
L precentral gyrus – L inferior temporal gyrus	-3.91	0.0418
L precentral gyrus – L olfactory cortex	-3.89	0.0418
L middle frontal gyrus – L amygdala	-3.89	0.0418
R precentral gyrus – L middle frontal gyrus, orbital part	-3.87	0.0432

L precentral gyrus – L parahippocampal gyrus	-3.87	0.0432
L superior frontal gyrus, dorsolateral – R temporal pole, middle temporal gyrus	-3.85	0.0441
L inferior frontal gyrus, orbital part – R superior frontal gyrus, medial	-3.82	0.0453
R inferior frontal gyrus, opercular part – R putamen	-3.81	0.0453
L middle frontal gyrus – L superior frontal gyrus, medial orbital	-3.8	0.0455
R middle frontal gyrus, orbital part – L postcentral gyrus	-3.78	0.0463
L middle frontal gyrus, orbital part – L supplementary motor area	-3.77	0.047
L middle frontal gyrus – L temporal pole, superior temporal gyrus	-3.76	0.047
R precentral gyrus – R inferior frontal gyrus, orbital part	-3.76	0.047
L rolandic operculum – L inferior occipital gyrus	-3.75	0.047
R rolandic operculum – L inferior occipital gyrus	-3.75	0.047
R temporal pole, superior temporal gyrus – L middle frontal gyrus	-3.75	0.047
L postcentral gyrus – L gyrus rectus	-3.73	0.0481
L temporal pole, superior temporal gyrus – R superior frontal gyrus, dorsolateral	-3.71	0.0494
**Apathy**		
L caudate – L thalamus	-5.89	0.0247
R caudate – R thalamus	-5.62	0.0247
R gyrus rectus – R parahippocampal gyrus	-5.41	0.0265
L caudate – R thalamus	-5.21	0.0322

L= left; R=right.

[a]*P* values are FDR corrected and adjusted to enforce monotonicity.

Supplementary Figure S1. Partial regression plots for a sample ROI-ROI functional connection in healthy controls.

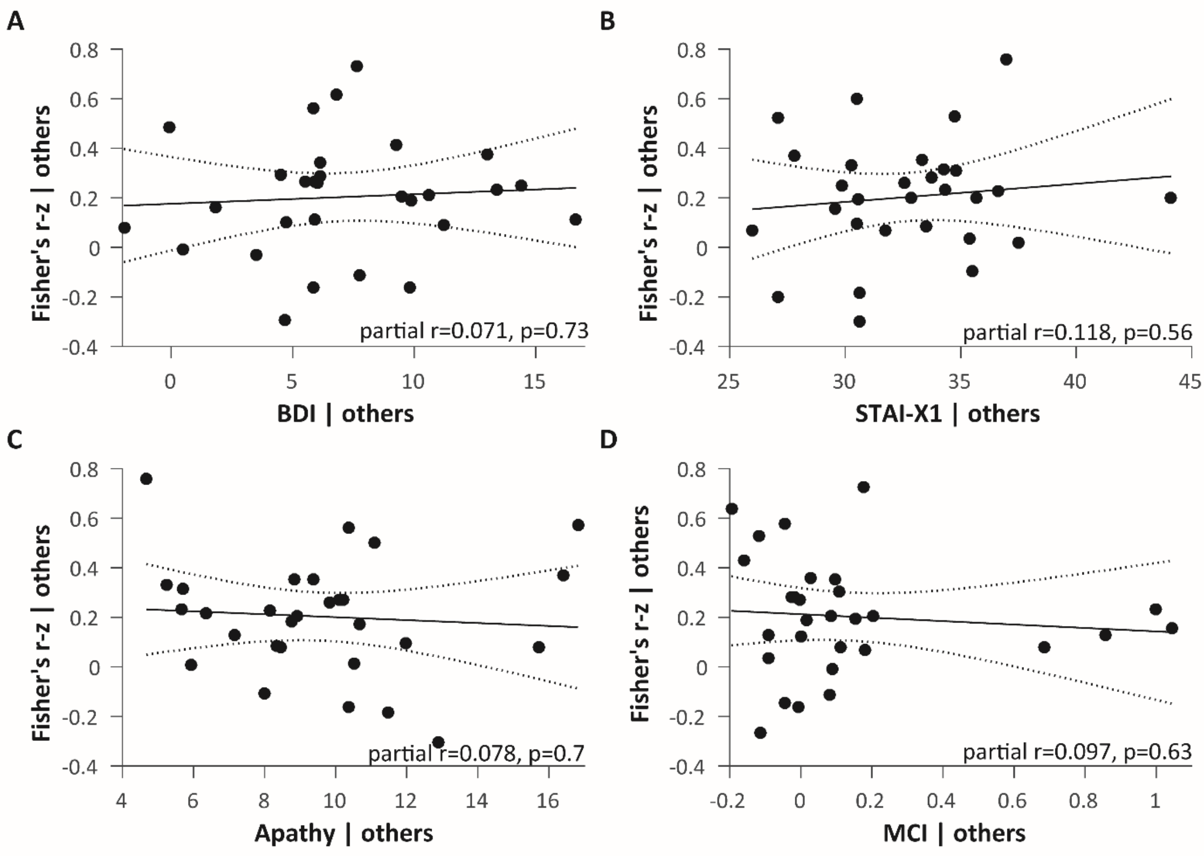

Partial regression plots are presented to illustrate in healthy controls the incremental effect of each neuropsychiatric scale on Fisher's transformed values by removing the effects of all other scales. The right gyrus rectus - right parahippocampal gyrus functional connection was chosen arbitrarily as an example due to its significant associations in PD patients with several of the neuropsychiatric scales. The associations are shown between Fisher's transformed values and **(A)** BDI-II; **(B)** STAI-state; **(C)** apathy and **(D)** MCI. The y-axis shows the residuals from regressing Fisher's transformed values against all neuropsychiatric scales other than the scale of interest, and the x-axis shows the residuals from regressing the scale of interest against all other neuropsychiatric scales. The linear fits are shown in solid lines and confidence intervals in dotted lines. Partial r = square root of coefficient of partial determination. None of the neuropsychiatric scales were significantly associated with the right gyrus rectus - right parahippocampal gyrus functional connection, or with any other functional connections in healthy controls.

Supplementary Table S2. Differences between PD patients and healthy controls in whole-brain functional connectivity.

ROI-ROI functional connection	*t* statistic (df=54)	*P* value (FDR corrected)[a]
**Healthy controls > PD patients**		
R rolandic operculum – L superior temporal gyrus	5.41	0.0051
R precentral gyrus – L postcentral gyrus	5.12	0.0057
L postcentral gyrus – R postcentral gyrus	5.07	0.0057
L rolandic operculum – R rolandic operculum	4.92	0.0067
R rolandic operculum – L paracentral lobule	4.89	0.0067
R rolandic operculum – R paracentral lobule	4.74	0.0074
L median cingulate gyrus – R temporal pole, superior gyrus	4.71	0.0074
R insula – L superior temporal gyrus	4.69	0.0074
L median cingulate gyrus – R superior parietal gyrus	4.69	0.0074
R median cingulate gyrus – R superior parietal gyrus	4.64	0.0078
L superior temporal gyrus – R superior temporal gyrus	4.61	0.008
R median cingulate gyrus – R temporal pole, superior gyrus	4.52	0.01
R rolandic operculum – R supplementary motor area	4.4	0.0136
R supplementary motor area – L superior temporal gyrus	4.37	0.0136
R insula – L superior parietal gyrus	4.36	0.0136
R inferior frontal gyrus, triangular part – L inferior parietal gyrus	4.33	0.0139
R precentral gyrus – L rolandic operculum	4.29	0.0139
L superior parietal gyrus – R putamen	4.29	0.0139
R inferior parietal gyrus – R inferior frontal gyrus, triangular part	4.29	0.0139
R superior temporal gyrus – L superior occipital gyrus	4.26	0.0146
L superior temporal gyrus – L median cingulate gyrus	4.23	0.0147
R insula – R precuneus	4.22	0.0147
R rolandic operculum – L superior parietal gyrus	4.2	0.0154
L superior temporal gyrus – R precuneus	4.17	0.016
L superior temporal gyrus – R precentral gyrus	4.15	0.0167
L superior temporal gyrus – R paracentral lobule	4.12	0.0178
R superior temporal gyrus – R fusiform	4.07	0.0195
L rolandic operculum – R supplementary motor area	4.06	0.0195
L rolandic operculum – R paracentral lobule	4.06	0.0195
R precuneus – R temporal pole, superior gyrus	4.04	0.02
R rolandic operculum – L median cingulate gyrus	4.01	0.0205
R postcentral gyrus – L paracentral lobule	4	0.0205
R putamen – L precuneus	4	0.0205
R precentral gyrus – R rolandic operculum	3.98	0.0207
R superior occipital gyrus – R superior temporal gyrus	3.98	0.0207
L superior occipital gyrus – R rolandic operculum	3.96	0.0215
R insula – R superior parietal gyrus	3.95	0.0217
R insula – L precuneus	3.94	0.0217
R precuneus – R superior temporal gyrus	3.92	0.0223
R precuneus – R putamen	3.91	0.0223
R superior occipital gyrus – R putamen	3.91	0.0223
L superior temporal gyrus – L paracentral lobule	3.89	0.0223
L superior temporal gyrus – L superior occipital gyrus	3.89	0.0223
L supplementary motor area – L inferior frontal gyrus, opercular part	3.88	0.0223

L superior temporal gyrus – L rolandic operculum	3.87	0.0223
R superior temporal gyrus – R insula	3.87	0.0223
R superior temporal gyrus – L lingual gyrus	3.84	0.0232
L rolandic operculum – L paracentral lobule	3.84	0.0232
L postcentral gyrus – R putamen	3.84	0.0232
R superior temporal gyrus – L insula	3.83	0.0232
L superior temporal gyrus – R median cingulate gyrus	3.82	0.0234
R supplementary motor area – L inferior frontal gyrus, opercular part	3.8	0.0249
R superior temporal gyrus – L median cingulate gyrus	3.78	0.0257
L superior occipital gyrus – R insula	3.78	0.0257
R insula – L rolandic operculum	3.77	0.0257
R insula – L insula	3.73	0.0278
L rolandic operculum – R postcentral gyrus	3.72	0.0278
R rolandic operculum – R superior parietal gyrus	3.71	0.0278
R putamen – L median cingulate gyrus	3.71	0.0278
L precentral gyrus – R precentral gyrus	3.71	0.0278
R supramarginal gyrus – R inferior frontal gyrus, triangular part	3.71	0.0278
L precentral gyrus – R putamen	3.71	0.0278
L median cingulate gyrus – R insula	3.7	0.0278
L median cingulate gyrus – L rolandic operculum	3.7	0.0279
L putamen – R angular gyrus	3.69	0.028
R pallidum – L superior parietal gyrus	3.69	0.028
L insula – R rolandic operculum	3.68	0.0283
L postcentral gyrus – R temporal pole, superior gyrus	3.66	0.0294
L putamen – L superior parietal gyrus	3.66	0.0294
L median cingulate gyrus – L superior parietal gyrus	3.64	0.0295
L rolandic operculum – R median cingulate gyrus	3.64	0.0295
L rolandic operculum – R superior temporal gyrus	3.64	0.0295
R rolandic operculum – R median cingulate gyrus	3.64	0.0295
R median cingulate gyrus – L superior parietal gyrus	3.63	0.0298
L insula – R precuneus	3.62	0.0305
L putamen – R superior occipital gyrus	3.6	0.0316
L supramarginal gyrus – R inferior frontal gyrus, opercular part	3.58	0.0328
R superior parietal gyrus – L putamen	3.58	0.0328
L superior parietal gyrus – L superior temporal gyrus	3.58	0.0328
L inferior parietal gyrus – R putamen	3.57	0.0328
L supramarginal gyrus – R supramarginal gyrus	3.57	0.0328
L superior temporal gyrus – R postcentral gyrus	3.56	0.0334
L middle occipital gyrus – R rolandic operculum	3.55	0.0334
R superior temporal gyrus – R supplementary motor area	3.54	0.0334
L superior temporal gyrus – R superior occipital gyrus	3.54	0.0334
R rolandic operculum – R precuneus	3.54	0.0334
R superior temporal gyrus – R median cingulate gyrus	3.53	0.0339
R superior temporal gyrus – R lingual gyrus	3.53	0.0339
L median cingulate gyrus – L olfactory cortex	3.52	0.0347
L postcentral gyrus – R insula	3.51	0.0352
R putamen – L paracentral lobule	3.49	0.0374
R inferior frontal gyrus, opercular part – L inferior frontal gyrus, opercular part	3.48	0.0381
R anterior cingulate gyrus – R angular gyrus	3.47	0.0382

R rolandic operculum – R lingual gyrus	3.46	0.0388
R temporal pole, middle gyrus – L temporal pole, middle gyrus	3.46	0.0388
R temporal pole, superior gyrus – L precuneus	3.45	0.04
R anterior cingulate gyrus – L fusiform	3.44	0.04
L superior temporal gyrus – L precuneus	3.44	0.04
R rolandic operculum – L postcentral gyrus	3.43	0.0411
R superior occipital gyrus – R insula	3.4	0.0442
R supplementary motor area – R insula	3.39	0.0446
L superior occipital gyrus – R putamen	3.39	0.0449
L paracentral lobule – R fusiform	3.39	0.0449
R temporal pole, superior gyrus – R supplementary motor area	3.38	0.0453
R superior temporal gyrus – R superior parietal gyrus	3.37	0.0462
L superior occipital gyrus – R rolandic operculum	3.36	0.0464
R putamen – L supplementary motor area	3.36	0.0464
R temporal pole, superior gyrus – R precentral gyrus	3.36	0.0464
R temporal pole, superior gyrus – R superior occipital gyrus	3.35	0.0464
R temporal pole, superior gyrus – L middle occipital gyrus	3.35	0.0464
L paracentral lobule – R precentral gyrus	3.35	0.0469
R median cingulate gyrus – L olfactory cortex	3.34	0.0469
R supplementary motor area – L insula	3.34	0.0469
R superior parietal gyrus – R inferior frontal gyrus, triangular part	3.33	0.0471
L precentral gyrus – R insula	3.33	0.0471
R supramarginal gyrus – L inferior frontal gyrus, opercular part	3.32	0.0477
L paracentral lobule – R insula	3.32	0.0477
R middle frontal gyrus – L lingual gyrus	3.31	0.0481
L median cingulate gyrus – L insula	3.31	0.0484
R superior occipital gyrus – R rolandic operculum	3.31	0.0484
R pallidum – L superior frontal gyrus, dorsolateral	3.3	0.0484
**PD patients > Healthy controls**		
R anterior cingulate gyrus – R caudate	3.34	0.0469

L= left; R=right.
[a]*P* values are FDR corrected and adjusted to enforce monotonicity.

Supplementary Methods. Neuropsychological battery used to assess cognitive function of Parkinson's disease patients.

The neuropsychological battery consisted of ten tests in the following five domains, with one measure from each test derived as recommended for cognitive evaluation in Parkinson's disease[1]. The following tests were used:

(1) Attention and working memory: Trail Making Test, part A[2] and Digit span backwards from the Wechsler Adult Intelligence Scale, third revision (WAIS-III)[3].

(2) Executive function: Tower of London[4] and semantic verbal fluency[5].

(3) Language: Boston Naming Test, Czech version[6,7] and WAIS-III Similarities[3].

(4) Memory: Rey Auditory Verbal Learning Test, delayed recall[8] and Brief Visuospatial Memory Test, revised, delayed recall[9].

(5) Visuospatial function: CLOX[10] and Judgment of Line Orientation[11].

Supplementary Table S3. Results of the neuropsychological evaluation as a function of cognitive domain.

Cognitive domain and tasks	PD patients (Mean ± SD)	Healthy controls (Mean ± SD)
**Attention and working memory**		
Trail Making Test, part A (TMT-A)	57.9±26.2	35.2±9.1
Digit Span Backwards (DS back)	6.0±2.3	6.7±2.2
**Executive function**		
Tower of London (ToL)	21.8±5.2	25.9±3.8
Semantic fluency: animals + clothes + shopping (COWAT)	59.1±13.6	65.8±11.8
**Language**		
Boston Naming Test, Czech version (BNT-60)	52.8±5.4	54.5±6.4
Wechsler Adult Intelligence Scale, third revision, similarities (WAIS-III Sim)	24.1±6.2	23.7±5.7
**Short term memory**		
Rey Auditory Verbal Learning Test, Delayed Recall (RAVLT-DR)	7.0±3.3	8.9±2.4
Brief Visuospatial Memory Test, Revised, Delayed Recall (BVMT-R-DR)	7.7±3.1	10.2±1.7
**Visuospatial function**		
Royall's CLOX (CLOX I)	12.1±2.5	13.2±1.1
Judgment of Line Orientation (JoL)	23.8±6.0	24.6±3.8

The raw scores (before transformation into a z-score) are provided for all tests.

Supplementary Table S4. Functional connections predicted by depression, anxiety and apathy in PD patients. Same analysis as in Supplementary Table S1 with the primary auditory and visual regions included.

ROI-ROI functional connection	*t* statistic (df=21)	*P* value (FDR corrected)[a]
**Depression**		
R gyrus rectus – R parahippocampal gyrus	5.6	0.0527
R anterior cingulate – R supramarginal gyrus	5.18	0.0527
L caudate – R thalamus	5.18	0.0527
R inferior frontal gyrus, opercular part – R angular gyrus	5.01	0.0553
L caudate – L thalamus	4.72	0.0553
L gyrus rectus – R parahippocampal gyrus	4.71	0.0553
R gyrus rectus – R hippocampus	4.7	0.0553
L anterior cingulate – R supramarginal gyrus	4.69	0.0553
R inferior frontal gyrus, triangular part – R posterior cingulate	4.69	0.0553
**Anxiety**		
Positive associations		
L gyrus rectus – R parahippocampal gyrus	8.64	0.0001
R gyrus rectus – R parahippocampal gyrus	7.36	0.0006
L gyrus rectus – L parahippocampal gyrus	5.38	0.0169
L subcallosal area – L superior frontal gyrus, medial orbital part	5.35	0.0169
R gyrus rectus – L superior frontal gyrus, orbital part	5.3	0.0169
L amygdala – R middle temporal gyrus	5.24	0.0169
R gyrus rectus – L parahippocampal gyrus	5.18	0.0169
L hippocampus – R middle temporal gyrus	5.12	0.0169
L gyrus rectus – R subcallosal area	5.07	0.0169
R gyrus rectus – L inferior temporal gyrus	4.91	0.0178
R amygdala – R middle temporal gyrus	4.9	0.0178
L gyrus rectus – L inferior temporal gyrus	4.79	0.0219
L gyrus rectus – R hippocampus	4.67	0.0256
L gyrus rectus – R amygdala	4.64	0.0256
L parahippocampal gyrus – L inferior frontal gyrus, orbital part	4.56	0.0268
R hippocampus – R middle temporal gyrus	4.55	0.0268
L olfactory cortex – L superior frontal gyrus, orbital part	4.52	0.0268
L olfactory cortex – L gyrus rectus	4.52	0.0268
L olfactory cortex – R superior frontal gyrus, medial orbital part	4.47	0.0275
L hippocampus – L superior frontal gyrus, orbital part	4.39	0.0302
L inferior temporal gyrus – L superior frontal gyrus, orbital part	4.37	0.0302
L hippocampus – L gyrus rectus	4.35	0.0306
R olfactory cortex – R gyrus rectus	4.23	0.0344
R amygdala – R gyrus rectus	4.19	0.0357
L parahippocampal gyrus – L superior frontal gyrus, orbital part	4.17	0.0357
L gyrus rectus – L superior frontal gyrus, orbital part	4.08	0.0407
L gyrus rectus – R gyrus rectus	4.02	0.0446
R parahippocampal gyrus – R middle temporal gyrus	3.95	0.048
R gyrus rectus – R middle temporal gyrus	3.94	0.048
R amygdala – R middle temporal gyrus	3.92	0.048
L parahippocampal gyrus – L middle frontal gyrus, orbital part	3.91	0.048
R inferior temporal gyrus – L superior frontal gyrus, orbital part	3.9	0.048

Negative associations		
L precentral gyrus – L superior frontal gyrus, orbital part	-5.48	0.0169
R amygdala – L superior frontal gyrus, dorsolateral	-5.18	0.0169
R precentral gyrus – L superior frontal gyrus, orbital part	-5.03	0.0169
L precentral gyrus – L inferior frontal gyrus, orbital part	-5.02	0.0169
L superior frontal gyrus, dorsolateral – L inferior frontal gyrus, orbital part	-5.01	0.0169
L postcentral gyrus – L middle frontal gyrus, orbital part	-4.94	0.0178
L precentral gyrus – L middle frontal gyrus, orbital part	-4.76	0.0221
R amygdala – L middle frontal gyrus	-4.66	0.0256
L rolandic operculum – R inferior occipital gyrus	-4.61	0.0262
L inferior frontal gyrus, orbital part – L middle frontal gyrus	-4.57	0.0268
R parahippocampal gyrus – L middle frontal gyrus	-4.5	0.0268
L postcentral gyrus – L superior frontal gyrus, orbital part	-4.49	0.0268
R superior frontal gyrus, dorsolateral – L temporal pole, middle temporal gyrus	-4.42	0.0299
L superior frontal gyrus, dorsolateral – L temporal pole, superior temporal gyrus	-4.38	0.0302
R superior frontal gyrus, dorsolateral – R amygdala	-4.37	0.0302
R supplementary motor area – R superior frontal gyrus, medial	-4.34	0.0306
L supplementary motor area – L superior frontal gyrus, orbital part	-4.29	0.0326
R supplementary motor area – L caudate	-4.29	0.0326
R middle frontal gyrus – R insula	-4.28	0.0326
L superior frontal gyrus, dorsolateral – L amygdala	-4.26	0.0332
L middle frontal gyrus – R hippocampus	-4.23	0.0344
R supplementary motor area – L superior frontal gyrus, orbital part	-4.2	0.0357
L precentral gyrus – L gyrus rectus	-4.17	0.0357
L precentral gyrus – L amygdala	-4.17	0.0357
L precentral gyrus – R inferior frontal gyrus, orbital part	-4.12	0.039
L gyrus rectus – L supramarginal gyrus	-4.11	0.0397
L middle frontal gyrus – L superior frontal gyrus, medial orbital part	-4.09	0.0402
R precentral gyrus – R superior frontal gyrus, orbital part	-4.04	0.0441
L superior frontal gyrus, orbital part – R inferior frontal gyrus, opercular part	-4.02	0.0446
R superior frontal gyrus, dorsolateral – L middle temporal gyrus	-3.98	0.0477
L supplementary motor area – L caudate	-3.95	0.048
R paracentral lobule – R superior frontal gyrus, medial part	-3.94	0.048
R inferior frontal gyrus, orbital part – L supramarginal gyrus	-3.94	0.048
L postcentral gyrus – L parahippocampal gyrus	-3.93	0.048
L precentral gyrus – R parahippocampal gyrus	-3.92	0.048
L precentral gyrus – L inferior temporal gyrus	-3.91	0.048
L precentral gyrus – L olfactory cortex	-3.89	0.048
L middle frontal gyrus – L amygdala	-3.89	0.048
R precentral gyrus – L middle frontal gyrus, orbital part	-3.87	0.0496
L precentral gyrus – L parahippocampal gyrus	-3.87	0.0496
**Apathy**		
L caudate – L thalamus	-5.89	0.0284
R caudate – R thalamus	-5.62	0.0284
R gyrus rectus – R parahippocampal gyrus	-5.41	0.0305

L caudate – R thalamus	-5.21	0.0370

L= left; R=right.
[a]P values are FDR corrected and adjusted to enforce monotonicity.

As shown in the table, the primary auditory and visual regions were not associated with any of the neuropsychiatric symptoms. Inclusion of these regions in the analysis did not introduce any qualitative changes in the findings for any of the neuropsychiatric symptoms.

References

1. Litvan, I. *et al.* Diagnostic criteria for mild cognitive impairment in Parkinson's disease: Movement Disorder Society Task Force guidelines. *Mov. Disord.* **27,** 349–56 (2012).
2. Bezdicek, O. *et al.* Czech version of the Trail Making Test: normative data and clinical utility. *Arch. Clin. Neuropsychol.* **27,** 906–14 (2012).
3. Wechsler, D. Wechsler Adult Intelligence Scale- 3rd edition. (WAIS-3). *San Antonio, TX Harcourt Assess.* (1997).
4. Michalec, J. *et al.* Standardization of the Czech version of the Tower of London test - Administration, scoring, validity. *Ces. a Slov. Neurol. a Neurochir.* **77,** 596–601 (2014).
5. Nikolai, T. *et al.* Tests of Verbal Fluency, Czech Normative Study in Older Patients. *Česká a Slov. Neurol. a Neurochir.* **78,** 292–299 (2015).
6. Tombaugh, T. N. & Hubley, A. M. The 60-item Boston Naming Test: norms for cognitively intact adults aged 25 to 88 years. *J. Clin. Exp. Neuropsychol.* **19,** 922–32 (1997).
7. Zemanová, N. *et al.* Validity Study of the Boston Naming Test Czech Version. *Česká a Slov. Neurol. a Neurochir.* **79,** 307–316 (2016).
8. Bezdicek, O. *et al.* Czech version of Rey Auditory Verbal Learning test: normative data. *Neuropsychol. Dev. Cogn. B. Aging. Neuropsychol. Cogn.* **21,** 693–721 (2014).
9. Benedict, R. H., Schretlen, D., Groninger, L., Dobraski, M. & Shpritz, B. Revision of the Brief Visuospatial Memory Test: Studies of normal performance, reliability, and validity. *Psychol. Assess.* **8,** 145–153 (1996).
10. Royall, D. R., Cordes, J. A. & Polk, M. CLOX: an executive clock drawing task. *J. Neurol. Neurosurg. Psychiatry* **64,** 588–594 (1998).
11. Woodard, J. L. *et al.* Normative data for equivalent, parallel forms of the Judgment of Line Orientation Test. *J. Clin. Exp. Neuropsychol.* **20,** 457–462 (1998).
